# Clinical and histological presentations of experimental autoimmune myocarditis in rats using different immunization schemes

**DOI:** 10.1186/1532-429X-16-S1-P282

**Published:** 2014-01-16

**Authors:** Sarah Jeuthe, Patrick Schmerler, Darach Oh-Ici, Katharina Wassilew, Dilyara Lauer, Susanne Mueller, Titus Kuehne, Felix Berger, Ulrike M Steckelings, Ludovit Paulis, Daniel Messroghli

**Affiliations:** 1 Congenital Heart Disease and Pediatric Cardiology, German Heart Institute Berlin, Berlin, Germany; 2 Pathology, German Heart Institute Berlin, Berlin, Germany; 3 Center for Cardiovascular Research, Charité-University Medicine, Berlin, Germany; 4 Experimental Neurology, Charité-University Medicine, Berlin, Germany; 5 Department of Cardiovascular and Renal Research, University of southern Denmark, Odense, Denmark; 6 Institute of Pathophysiology, Comenius University, Bratislava, Slovakia

## Background

Myocarditis is an inflammatory cardiac disease with a wide range of symptoms and various etiologies. The diagnostic workup and the treatment of myocarditis remain highly controversial. Therefore, there is a high demand for reliable animal models. Experimental autoimmune myocarditis (EAM) represents a model of human myocarditis and heart failure. In this study we investigated the histological, functional and clinical presentations of EAM induced by different immunization schemes.

## Methods

Male young Lewis rats were divided into 5 groups immunized by porcine myocardial myosin: 1 mg subcutaneously (SC) on day 0 and 7; 0.25 mg subcutaneously into rear footpads (RF) on day 0; 0.5 mg RF on day 0; and 1 mg RF on day 0; and a sham group, injected with a vehicle solution without myosin into the RF on day 0. Rats were sacrificed on day 21 after assessment of left ventricular (LV) function by cardiac magnetic resonance imaging and cardiac catheterization under isoflurane anesthesia. The ventricle weight was measured and the location, type and degree of myocardial inflammatory infiltrates were determined by conventional histology and immunohistochemistry (CD 68).

## Results

Rats immunized in the RF showed a mortality of 20%, 20% and 44% for the 0.25 mg, 0.5 mg and 1 mg myosin doses respectively, while the SC group and the RF sham group yielded a mortality of 0%. Morbidity as defined by inflammatory infiltrates on HE staining was 22% in the SC immunized rats, 0% in the RF sham group and 100% in all actively RF immunized groups. Histologically we observed macrophage-rich inflammatory infiltrates in both ventricles. All RF groups immunized with myosin showed augmented relative ventricle weight and spleen weight, increased LV end-diastolic pressure, reduced LV developed pressure and reduced LV ejection fraction, without any dose-dependend effect.

## Conclusions

Subcutaneous immunization into the neck and flanks failed to induce a reproducible EAM, while RF administration of myosin reliably led to EAM. Low-dose RF myosin application induces the complete histological and clinical picture of EAM, associated with lower mortality, non-specific symptoms and animal distress.

## Funding

Sarah Jeuthe, this work is supported by a research grant of the German Cardiac Society (DGK).

